# Super-Resolution Dynamic Imaging of Dendritic Spines Using a Low-Affinity Photoconvertible Actin Probe

**DOI:** 10.1371/journal.pone.0015611

**Published:** 2011-01-17

**Authors:** Ignacio Izeddin, Christian G. Specht, Mickaël Lelek, Xavier Darzacq, Antoine Triller, Christophe Zimmer, Maxime Dahan

**Affiliations:** 1 Laboratoire Kastler Brossel, CNRS UMR 8552, Departments of Physics and Biology, École Normale Supérieure, Université Pierre et Marie Curie-Paris 6, Paris, France; 2 Biologie Cellulaire de la Synapse, École Normale Supérieure, Inserm U1024, Paris, France; 3 Institut Pasteur, Groupe Imagerie et Modélisation, CNRS, URA 2582, Paris, France; 4 Régulation de l'Expression Génétique, École Normale Supérieure, CNRS UMR8541, Paris, France; University of California, United States of America

## Abstract

The actin cytoskeleton of dendritic spines plays a key role in morphological aspects of synaptic plasticity. The detailed analysis of the spine structure and dynamics in live neurons, however, has been hampered by the diffraction-limited resolution of conventional fluorescence microscopy. The advent of nanoscopic imaging techniques thus holds great promise for the study of these processes. We implemented a strategy for the visualization of morphological changes of dendritic spines over tens of minutes at a lateral resolution of 25 to 65 nm. We have generated a low-affinity photoconvertible probe, capable of reversibly binding to actin and thus allowing long-term photoactivated localization microscopy of the spine cytoskeleton. Using this approach, we resolve structural parameters of spines and record their long-term dynamics at a temporal resolution below one minute. Furthermore, we have determined changes in the spine morphology in response to pharmacologically induced synaptic activity and quantified the actin redistribution underlying these changes. By combining PALM imaging with quantum dot tracking, we could also simultaneously visualize the cytoskeleton and the spine membrane, allowing us to record complementary information on the morphological changes of the spines at super-resolution.

## Introduction

Fluorescence microscopy using genetically encoded fluorescent proteins has greatly advanced our understanding of many functional biological systems over the last decade. However, the precision at which cellular structures can be visualized has been limited by the spatial resolution imposed by the diffraction limit of light (∼250 nm). Novel super-resolution imaging methods using photoactivatable proteins or photoswitchable fluorophores bypass this limitation and have the potential to revolutionize the experimental scope of light microscopy [1]. The application of these methods in living cells is far from trivial, although recent work has achieved a remarkable progress in this direction [2]–[7]. A general problem is the time needed to acquire a super-resolution image and the associated bleaching of fluorophores, which limits the temporal resolution as well as the use of these techniques for long-term imaging.

Dendritic spines are small cellular structures that compartmentalize the sites of excitatory neurotransmission in neurons [8]. The small dimensions of spines (∼500 nm in diameter) make super-resolution methods ideally suited to image the morphology of spines at much greater detail than that achieved by conventional fluorescence microscopy. The structure of dendritic spines is defined by the F-actin cytoskeleton and can undergo fast dynamic morphological changes that are believed to contribute to the plasticity of synaptic transmission [9]. While physiological and morphological aspects of synaptic plasticity are under certain conditions independent from one another [10], the enhancement of synaptic transmission by long-term potentiation (LTP) appears to be generally associated with an increase in spine volume [4], [11]. In line with these observations, the polymerization state and the dynamic properties of the actin cytoskeleton in dendritic spines have been shown to change during synaptic plasticity [12], [13]. These studies aimed at identifying different actin pools by distinguishing between populations of actin molecules using photoactivatable fluorophores and FRET, respectively. Morphological changes associated with synaptic activity occur on relatively slow time scale of tens of minutes [4], however the tools to image these changes continuously at high spatial resolution are limited. Therefore the goal of our study was to develop a strategy that would enable us to visualize the dynamic changes of the spine morphology for long periods.

Recent studies have combined the use of photoactivatable probes and single particle tracking (SPT) to study the kinetics of the actin cytoskeleton of spines [14], [15]. The short-range motion of individual actin molecules in these experiments suggested a complex meshwork of actin filaments in dendritic spines, in line with observations made by electron microscopy (EM) [16], [17]. However, SPT-based experiments lack the strength of a direct visualization of the changes of the spine morphology. In this project, we have therefore chosen a very different approach. Rather than inferring the structure from the tracking of single molecules, we aimed at reconstructing the structure itself, meaning that we tried to visualize the sub-spine actin organization. To this aim, we have chosen to use photoactivated localization microscopy (PALM) [18], [19]. The principle of PALM is to sequentially activate, image and bleach a sparse subset of fluorescent molecules. By accurately localizing individual activated molecules in each frame, images can be obtained with sub-diffraction resolution. We have generated a probe that binds reversibly to actin, thus acting as a ‘scanner’ for the distribution of actin within spines. The low-affinity of this probe allows for the replenishment of the bleached fluorophores and thereby overcomes the limitation of long-term PALM imaging of fluorescently tagged fusion proteins. Here, we have used this actin probe to determine the morphology of individual spines and record their baseline dynamics and their evolution during pharmacologically induced synaptic activity.

## Results

### PALM imaging of the actin cytoskeleton using a low affinity photoconvertible probe

To study the organization of the actin cytoskeleton we designed an actin probe that combines an actin-binding peptide (ABP) sequence [20] and tandem Eos fluorescent protein (tdEosFP). This construct is designed to bind reversibly to the F-actin cytoskeleton and can be used for PALM imaging due to its photoconversion from a green to a red fluorescent form upon illumination with 405 nm light.

**Figure 1:**
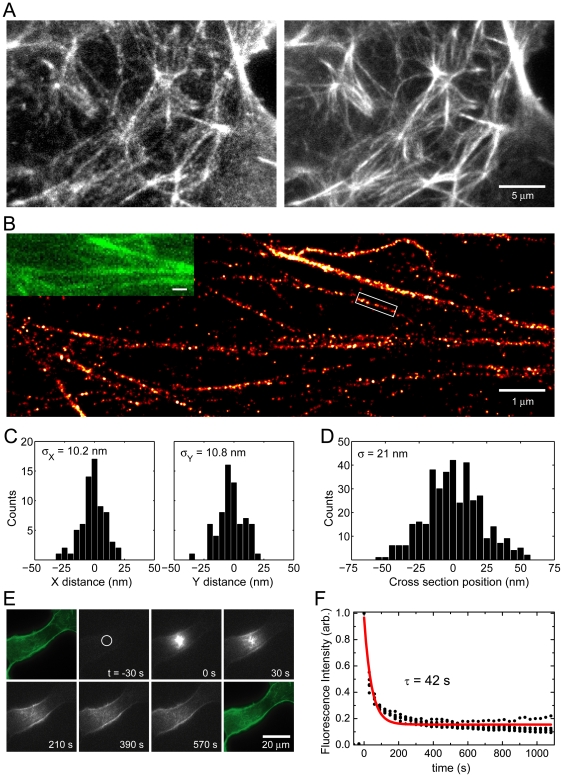
ABP-tdEosFP for actin labeling: spatial resolution and reversible binding.

When expressed in COS-7 cells, ABP-tdEosFP labels filamentous actin structures with high specificity, as judged by the colocalization with phalloidin labeled F-actin (Fig. 1A). The close correspondence between the two labels also suggests that ABP-tdEosFP has no preference for different pools of F-actin. Sparse subsets of individual ABP-tdEosFP molecules were photoconverted, imaged and bleached under continuous illumination with the 405 nm and 561 nm lasers. The position of each individual fluorophore was determined by fitting the point spread function (PSF) of their fluorescent signal to a two-dimensional Gaussian distribution. By superimposing the positions of all the detected fluorophores a super-resolution image was obtained, in which the actin cytoskeleton can be seen in much greater detail (Fig. 1B). This revealed that some of the observed thick fibers are clearly composed of several thinner structures, while others represent densely packed bundles of actin filaments.

The spatial resolution of PALM imaging depends on the ability to accurately determine the position of each single molecule, and ultimately on the number of detected photons emitted by the fluorophore, for EosFP typically in the range of hundreds of photons [21]. In order to experimentally determine the resolution of our system, we analyzed the spatial distribution of the positions from isolated ABP-tdEosFP fluorophores, recorded in consecutive frames in a fixed sample (Fig. 1C). The standard deviation σ of the X and Y positions of isolated fluorophores ranges from 10 to 15 nm. The spatial resolution of an image that is reconstructed from individual molecules can be regarded as the full width at half maximum (FWHM) of the Gaussian curve representing the localization uncertainty σ. Therefore, in our case, the spatial resolution lies between 25 and 35 nm. Indeed, cross section profiles across filamentous actin structures reveal that these have a thickness of at least 50 nm, well above the limit of our spatial resolution (Fig. 1B,D). The structures are therefore likely to represent bundles of several actin filaments, since individual filaments are known to measure only 8–10 nm in diameter [17].

To confirm the reversibility of the binding of ABP-tdEosFP to F-actin we also performed photoactivation experiments in live COS-7 cells (Fig. 1E,F). A small area of the cell was activated with a brief 405 nm pulse, converting the local population of ABP-tdEosFP into the red form of the fluorophore. By time-lapse imaging we then followed the exchange of this pool of fluorophores with the unconverted bulk of the ABP-tdEosFP pool. We observed that the ABP-tdEosFP signal quickly diffused with a time constant of about 40 s.

Taken together, these experiments show that 1) ABP-tdEosFP has a high specificity for F-actin, 2) that its low affinity allows ABP-tdEosFP to exchange between different F-actin binding sites and 3) that PALM imaging of ABP-tdEosFP yields super-resolution images of the actin cytoskeleton down to a spatial resolution of ∼25 nm.

### Super-resolution imaging of dendritic spines in hippocampal neurons

**Figure 2:**
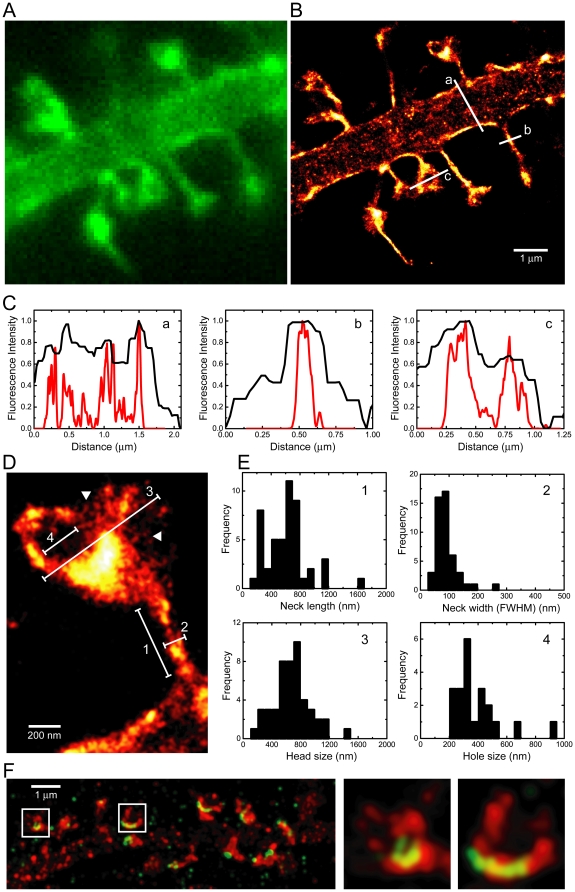
PALM super-resolution microscopy of dendritic spines. (A) Conventional fluorescence microscopy of a rat hippocampal neuron expressing ABP-tdEosFP and fixed at DIV 25 (unconverted form of the fluorophore). (B) Super-resolution PALM image of the same dendritic segment, acquired with a frame rate of 50 ms (8000 frames). (C) Profiles across the dendritic shaft (a), the spine neck (b), and the spine head (c), as indicated in (B). (D) Detail of a single dendritic spine. (E) Quantification of the spine parameters indicated in (D): neck length (panel 1; N = 48 spines from 4 cells and 3 independent experiments), neck width (2; N = 48, 4 cells, 3 experiments), spine head diameter (3; N = 48, 4 cells, 3 experiments), and diameter of actin-free regions (‘hole’, 4; N = 21, 4 cells, 3 experiments). (F) Two-color PALM/dSTORM reconstruction of the spine actin cytoskeleton (in red) in relation to the postsynaptic density protein Shank2 (green).

We then transfected rat hippocampal neurons with ABP-tdEosFP and fixed the cultures at 3 to 4 weeks *in vitro*. By this time, the neurons had formed dense networks and exhibited mature dendritic spines. As expected, ABP-tdEosFP was accumulated in spines as a result of the high local concentration of actin (Fig 2A). Long-term expression of ABP-tdEosFP did not affect the spine density (average of 13.1 spines per 10 µm dendrite, N = 8 fields of view from 3 independent experiments), which was found to be comparable to the synapse density of untransfected neurons of similar age [22].

Using PALM image reconstruction we obtained high-resolution images of mature dendritic spines (Fig. 2B). Dendritic protrusions displayed a great variety of shapes and sizes, and included thin, stubby, cup- or mushroom-shaped spines as well as filopodia (see Fig. S1). Given the high spatial resolution of PALM microscopy we could measure the dimensions of spines in great detail as revealed by intensity profiles across different dendritic structures (Fig. 2C). In this context it should be mentioned that our images represent a 2D projection of an optical slice with a depth of about 500 nm. To accurately measure the described parameters, we have therefore focused on spines protruding from the dendrite parallel to the focal plane. We measured the length and the width of the neck of thin, cup- and mushroom-shaped spines, as well as the width of the spine heads (Fig. 2D, E). The quantification of these data shows that the width of the spine neck is relatively homogeneous with about 90±40 nm (mean ± standard deviation, N = 48 spines from 3 independent experiments) in diameter. In contrast, the length of the spine neck was much more variable, ranging from about 250 to 800 nm (N = 48). The average width of the spine head was 670±250 nm (N = 48). These values are similar to those obtained by EM [23], [24]. We did not observe any correlation between the dimensions of the spine head and the length and thickness of the spine neck (Fig. S1), suggesting that these parameters are regulated independently [23]). The fact that our photometric measurements are in line with EM findings implies that long-term expression of ABP-tdEosFP does not affect the organization of the spine cytoskeleton. One of the limitations of EM, however, is that it cannot be applied to living cells, which is why PALM imaging has the great advantage that it can give access to dynamic processes at super-resolution, such as the temporal fluctuations of the spine morphology (see below).

Large spine heads were either cup-shaped or mushroom-shaped, in which case they frequently enclosed a region in which no ABP-tdEosFP signals were detected (Fig. 2C panel (c) and Fig. 2D). We hypothesized that this ‘hole’ represents a depression in the surface of the spine head viewed from above and possibly formed by a presynaptic bouton. The quantification of the cup-shaped structures and the holes gave a mean diameter of about 380±160 nm (N = 21; Fig. 2E), similar to the dimensions of the postsynaptic density (PSD) of 200–500 nm [25]. In order to characterize these actin-free regions, we labeled the PSD with a specific antibody against the scaffold protein Shank2 and a secondary antibody coupled with Alexa647. This fluorophore can exchange between off and on states under reducing conditions, thus lending itself to direct stochastic optical reconstruction microscopy (dSTORM) imaging [26]. Dual-color super-resolution imaging of mature dendritic spines revealed that Shank2 is located at the tip of the spines, overlaps only partially with the ABP-tdEosFP signal, and was occasionally associated with cup shapes as well as with actin-free areas of the spine head (Fig. 2F).

Closer inspection of the high-resolution PALM images shows that actin is not homogeneously distributed within the spine (arrowheads in Fig. 2D). Rather, we noticed a filamentous network that appeared to reticulate the spine head. This was most noticeable in the regions of the spine with a relatively low actin concentration. While the thickness of the observed structures is below the limit of our resolution, their length reached up to 100 nm.

### Optimization of long-term dynamic imaging

Given the role of the actin cytoskeleton in morphological aspects of synaptic plasticity [9], our next aim was to measure temporal changes of the structural parameters described above. We therefore adapted our strategy for super-resolution long-term dynamic recording of the dendritic spine morphology.

**Figure 3:**
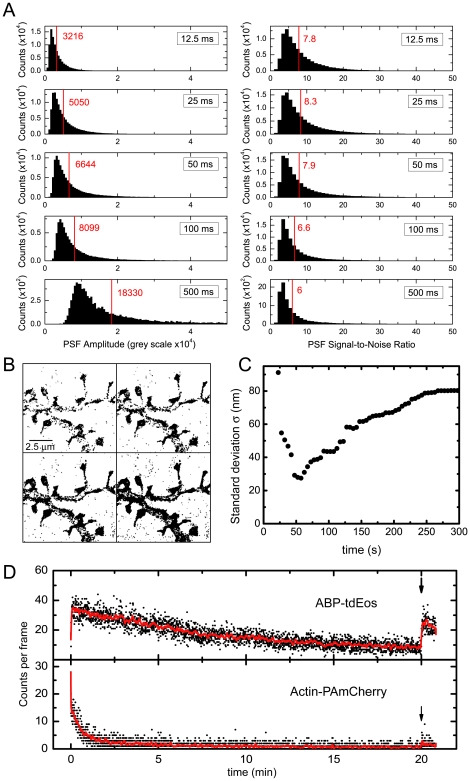
Super-resolution dynamics: spatial versus temporal resolution. (A) Optimization of the SNR as a function of the image acquisition time. The left panels show the histograms of the amplitudes of the recorded signals at different acquisition times (12.5 ms to 500 ms); the right panels show their respective SNRs. The mean values are indicated as red lines. (B) PALM reconstruction of fixed dendritic spines from 500, 1000, 2000, and 5000 frames of 25 ms. (C) Standard deviation of the distribution of signals across a thin filopodium from the live recording shown in Fig. S2, as a function of the time window used for image reconstruction. (D) Time course of the number of detected molecules for ABP-tdEosFP and actin-PAmCherry at constant illumination. The arrow indicates an increase of the photoconversion laser power at the end of the experiment (5× for ABP-tdEosFP, 10× for actin-PAmCherry).

In order to determine the best conditions for live PALM imaging, we adjusted the image acquisition time to optimize the signal-to-noise-ratio (SNR) of the individual fluorophores. To ensure the fastest possible sampling of the structures, we chose the maximal excitation laser power (561 nm at 4 kW/cm^2^) to bleach the activated fluorophores effectively. We then recorded individual fluorophores at different frame acquisition rates and measured their SNR (Fig. 3A). We found that the mean SNR was highest at 25 ms and 50 ms of acquisition (8.3 and 7.9, respectively). We therefore performed our live experiments consistently at 25 ms exposure time.

For the reconstruction of live super-resolution images the spatial and the temporal resolution are intimately correlated. The number of frames for the reconstruction of the PALM image (that determines the temporal resolution) must be adjusted such that the Nyquist-Shannon criterion is satisfied. This criterion states that the sampling frequency must be at least twice as fine as the desired spatial resolution [27]. Taking this into account, we measured the number of detected fluorophores per unit of time in a given area of our structures. We found that in different cellular compartments the typical density of detections within 2000 frames of 25 ms ranged from ρ = 2×10^−3^ to 8×10^−3^ nm^−2^, which results in a theoretical maximal spatial resolution of 44 to 90 nm (or 62 to 126 nm for 1000 frames) considering a constant density probability [28]. To test this concept, we reconstructed PALM images with 500, 1000, 2000, and 5000 frames of 25 ms from a fixed dendritic segment (Fig. 3B). Judging from these images, we found that 1000 to 2000 frames are sufficient to reach a density of reconstruction that satisfies the Nyquist-Shannon criterion.

We then imaged live hippocampal neurons expressing ABP-tdEosFP for periods of up to 30 min of constant illumination. Since cellular structures move during live experiments, the time required for the reconstruction of one PALM image must not exceed the time scale of the morphological changes. Hence, there is a compromise between the spatial and the temporal resolution of the recording. In order to determine the best conditions for PALM image reconstruction, i.e. the highest possible temporal and spatial resolution, we chose cultured neurons at day *in vitro* (DIV) 9. At this developmental stage, the dendritic network is still very immature and displays thin filopodia-like protrusions that undergo relatively fast movements (Fig. S2). In Figure 3C we measured the standard deviation of the recorded signals along a profile across a filopodium (from Fig. S2). As can be seen, there is an optimum reconstruction time of ∼50 s (2000 frames of 25 ms), with a standard deviation of 27 nm (spatial resolution of ∼65 nm). Choosing a larger number of frames for reconstruction reduces the spatial resolution of the image due to the cell dynamics, whilst a smaller number of frames has the same effect due to the low density of detected fluorophores. It is noteworthy that this measurement has been performed on a thin structure (≤65 nm) with a low density of single molecule events per image frame. In practice, this means that the chosen number of frames for PALM reconstruction not only depends on the biological system and photostability of the fluorophore, but also on the region of interest. In our case, 50 s (2000 frames) are necessary to image thin structures such as filopodia or spine necks, whereas 25 s (1000 frames) may be sufficient for the spine head.

During acquisition, ABP-tdEosFP molecules are continuously photoconverted, imaged and finally bleached, meaning that the pool of unconverted fluorophores is slowly depleted. This effect causes a reduction of the number of events that are detected per image frame at constant illumination (Fig. 3D). However, the reduction is relatively slow and can be compensated by an increase in the 405 nm laser intensity used for photoconversion of ABP-tdEosFP (see arrow in Fig. 3D), which permitted us to do long-term recordings (up to 30 min of constant illumination). In contrast, a β-actin construct tagged with photoactivatable mCherry [29] decayed much faster during continuous imaging and could not be compensated by an increase of the activation laser. The likely cause of this difference is the reversibility of the ABP-tdEosFP binding to actin that allows the replenishment of the fluorophore (Fig. 1E,F), in combination with the high number of binding sites for ABP-tdEosFP. It is noteworthy that only molecules attached to F-actin are being detected as well-defined PSF signals. Unbound ABP-tdEosFP or fluorophores attached to G-actin diffuse too rapidly to be detected with our 25 ms acquisition time [30], and therefore only contribute to the background noise.

In summary, ABP-tdEosFP serves as an ideal tool to achieve a high coverage of the actin cytoskeleton for relatively long periods of recording despite ongoing photobleaching. The reconstruction of PALM images from 2000 frames (50 s at 40 Hz) was generally sufficient to achieve a good sampling while providing an adequate temporal resolution to visualize the movements of dendritic spines or filopodia.

### Dynamic imaging of mature dendritic spines

**Figure 4:**
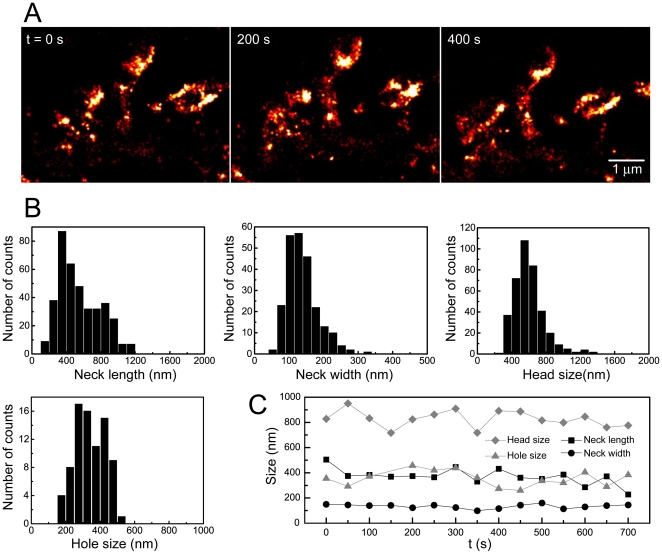
Spine dynamics in mature hippocampal neurons. (A) Super-resolution time-lapse imaging of dendritic spines from a neuron expressing ABP-tdEosFP at DIV 27. Each PALM image was reconstructed from 2000 frames recorded at 25 ms. (B) Quantification of morphological spine parameters (as in Fig. 2E) in living neurons (neck width: 140±45 nm mean ± standard deviation, N = 236 spines; neck length: 550±240 nm, N = 385; head diameter: 600±180 nm, N = 385; diameter of holes and cup-shapes: 340±85 nm, N = 81; from 12 cells and 3 independent experiments). (C) Baseline dynamics of the morphological parameters of an individual spine under control conditions.

Having established the best conditions for live PALM imaging, we then recorded the baseline dynamics of mature dendritic spines in hippocampal cultures at DIV 20 to 30 under reduced levels of synaptic activity (MEM-based imaging medium containing 1 µM TTX). These recordings were done at constant illumination for up to 15 minutes at 40 Hz and images were reconstructed from 2000 individual frames (Fig. 4A). For a smoother display, movies of the F-actin dynamics were generated using a temporal sliding window (see Movie S1).

We then quantified morphological parameters of the spine actin cytoskeleton in living neurons (Fig. 4B). The parameters that were measured are the same as those quantified in Fig. 2E. In living hippocampal neurons, the length of the neck varied from about 200 to 1000 nm, with a mean diameter of 140±45 nm (mean ± standard deviation, N = 236 spines from 3 independent experiments). The average width of the spine head was 600±180 nm (N = 385), while the diameter of the actin-free regions (holes) and the cup-shaped structures measured 340±85 nm (N = 81). Altogether, the spine parameters obtained in the live experiments are in line with the data from fixed neurons, suggesting that the fixation did not noticeably alter the spine cytoskeleton. We also measured the evolution of the morphology of individual spines over time (Fig. 4C). Interestingly, the width of the spine head, the size of the actin-free areas and the length of the neck varied substantially, whereas the thickness of the neck remained relatively constant. In order to quantify the variability of these parameters as a function of time, we calculated the standard deviation of the neck length and width, head size, and actin-free regions of individual spines during a period of 12 minutes; we then averaged the temporal standard deviation for all the dendritic spines and obtained 60 nm (N = 27) for the neck length, 24 nm (N = 17) for the neck width, 64 nm (N = 27) for the head size, and 71 nm (N = 7) for the hole size. These values represent the temporal fluctuations of the spine cytoskeleton under baseline conditions and show that the absolute change of the neck width is small compared to the other parameters.

### Dual super-resolution imaging of the spine cytoskeleton and plasma membrane

In the experiments described thus far we inferred the morphology of the spine from the distribution of the actin cytoskeleton as judged by the localization of the ABP-tdEosFP probe. We therefore sought to relate our measurements of the actin cytoskeleton to the position of the plasma membrane, in order to study how temporal changes of the actin cytoskeleton are translated into a modification of the spine membrane.

**Figure 5:**
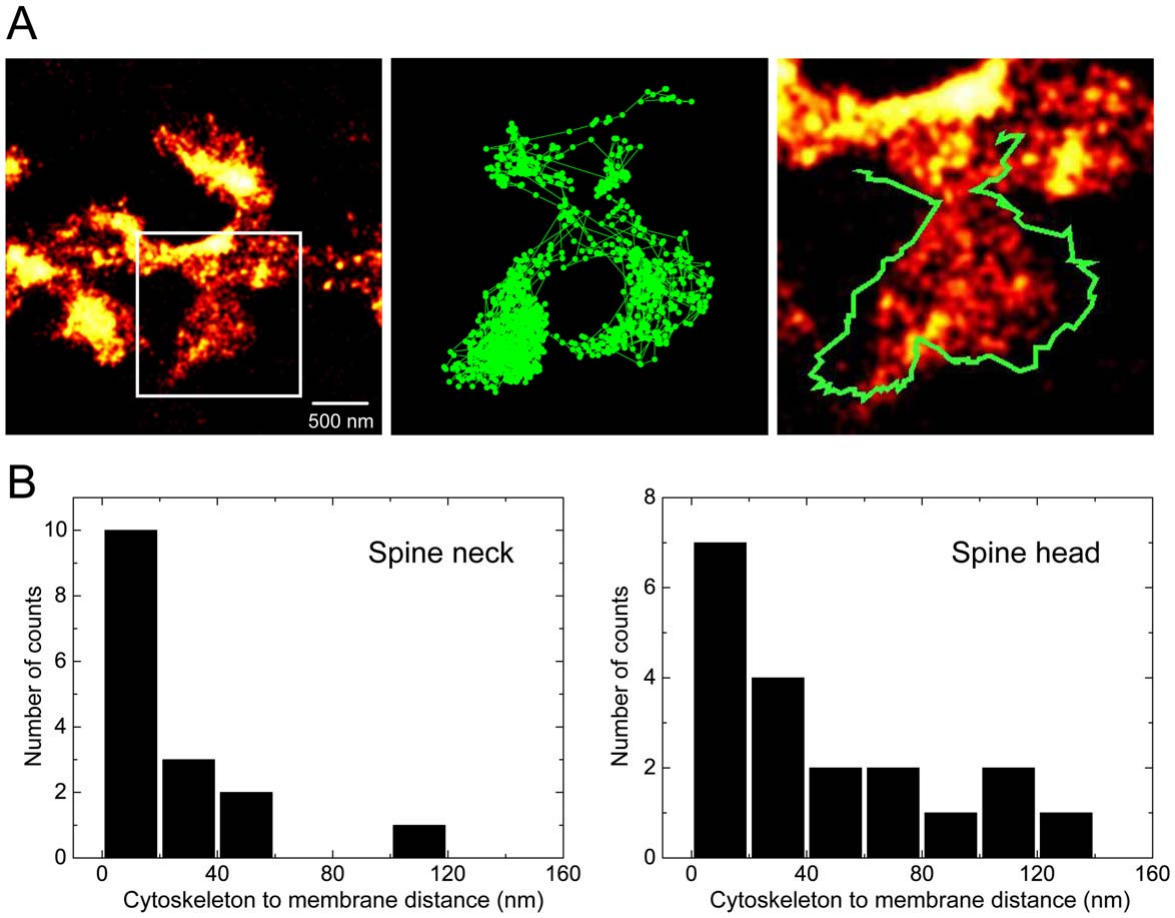
Dual super-resolution imaging of the spine membrane and the cytoskeleton. (A) PALM imaging of ABP-tdEosFP combined with QD tracking of a diffusing membrane construct (DIV 23). The QD trajectory is depicted as a green line (middle panel) and its outline is superimposed in the overlay with the actin cytoskeleton (right). Both images were reconstructed from 2000 frames recorded at 25 ms. (B) Measurement of the distance between the cytoskeleton and the membrane at the neck (left histogram, N = 16 spines from 9 cells and 2 independent experiments) and the spine head (right, N = 19 spines, 12 cells, 2 experiments).

We co-expressed a GFP-tagged membrane construct (GFP-GPI) together with ABP-tdEosFP in hippocampal neurons. By attaching quantum dots (QDs, 705 nm emission) to the extracellular GFP domain using specific antibodies, we could visualize, in parallel and with a localization accuracy below the diffraction limit of light, the ABP-tdEosFP labeling the actin cytoskeleton and the position of the QDs at the cell membrane. The lateral diffusion of the QD-tagged GPI was determined by single particle tracking (SPT) and served as a readout for the plasma membrane (Fig. 5A, Movie S2).

We could then measure the distance between the plasma membrane outlined by the QD trajectories and the actin cytoskeleton, as reconstructed by the PALM images (Fig. 5B). In both the neck and the spine head, this was achieved by measuring the diameter across the structure in the two channels and dividing the difference by two. The diffusion of the QDs within the spine determined the sampling rate at which dual images could be reconstructed; typically 2000 frames of 25 ms were enough to obtain a complete outline of the plasma membrane. It should be kept in mind that QD diffusion may be restricted in crowded membrane compartments such as synapses, and that the QDs may thus not always explore the entire membrane surface (see Fig. 5A). Furthermore, the QDs used in this study are relatively large (∼25 nm). We have therefore excluded spines with incomplete QD sampling from the analysis.

We found that in the spine neck, the distance between the two structures was generally below 50 nm (24±28 nm, mean ± standard deviation, N = 16); in contrast, in the spine head we observed a larger apparent distance between the cytoskeleton and the membrane (46±40 nm, N = 19). Nonetheless, in most of the measured spine heads the membrane was within 50 nm of the cytoskeleton. These data show that the super-resolution imaging of the actin cytoskeleton is an accurate readout of the spine morphology. Although both approaches are to a certain extent complementary, our measurements of the actin cytoskeleton provide additional information on the internal organization of synaptic spines.

### Activity-dependent remodeling of the dendritic spine cytoskeleton

The strength of synaptic neurotransmission is regulated by activity-dependent processes, and includes morphological alterations of dendritic spines. These dynamic changes are brought about by rearrangements of the actin cytoskeleton [9]. To investigate the early effects of synaptic activity on the actin distribution within dendritic spines, we performed live PALM imaging during pharmacological activation of AMPA (α-amino-3-hydroxy-5-methyl-4-isoxazolepropionic acid) receptors. In these experiments, the glutamate receptor agonist AMPA was added to the bath after recording of the baseline dynamics, and remained present throughout the measurement (up to 10 min).

**Figure 6:**
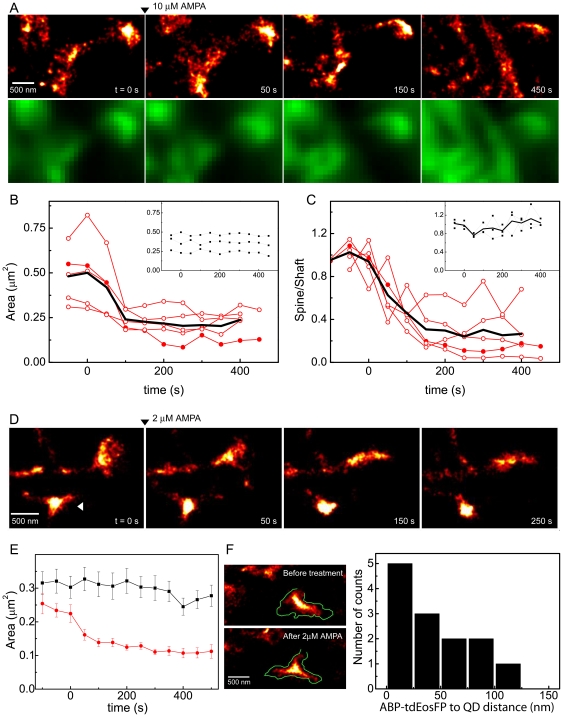
Structural dynamics of synaptic spines during AMPAR activation. (A) Upper panels: super-resolution time-lapse imaging of a single spine from a DIV 28 neuron expressing ABP-tdEosFP. AMPA was added at t = 0 at a final concentration of 10 µM (arrowhead). Lower panels: average projection of the raw frames used for PALM reconstruction. (B) Quantification of the change of the area of the spine heads over time. (C) Redistribution of the actin cytoskeleton in response to the bath application of AMPA: ratio of number of counts in the spine head relative to the counts in the shaft of the spine. In both, (B) and (C), full circles represent the spine shown in panel (A) and the black line represents the averaged data (N = 5 spines from 3 cells and 3 independent experiments). In the insets to the graphs, measured control spines without treatment (N = 3 spines, 2 cells, 2 experiments). (D) PALM imaging of spines during bath application of 2 µM AMPA (DIV 27), the arrowhead indicates a spine that rounds up after treatment. (E) Quantification of the change of the area of the spine heads over time under control conditions (N = 22 spines from 6 cells and 3 independent experiments) and 2 µM AMPA treatment (35 spines, 4 cells, 2 experiments). (F) Simultaneous PALM/QD imaging of the spine cytoskeleton and the plasma membrane, before and after application of 2 µM AMPA. The outline of the membrane is superimposed as a green trace to the PALM image (left). Histogram of the distance between the cytoskeleton and the spine head during bath application of 2 µM AMPA (right, N = 13 spines, 2 cells, 2 independent experiments).

In response to the application of 10 µM AMPA we observed systematic changes in the distribution of actin within the spine head (Fig. 6A upper panels, Movies S3, S4). Within approximately 2 min after application, the central regions of the spine head were more enriched in ABP-tdEosFP at the expense of the peripheral regions of the spine head. As a result, the size of the spine head was drastically reduced after AMPA application. Concurrently, the spine head lost actin molecules during AMPAR activation, as judged by a reduction in the ABP-tdEosFP fluorophore detections in the spine head over time and the increase of the actin levels in the shaft. However, in the PALM images only the bound pool of ABP-tdEosFP contributes to the image reconstruction, since these fluorophores produce well-defined PSF signals. Nevertheless, the actin redistribution was also apparent in the diffraction-limited images, where the diffuse pool of ABP-tdEosFP contributes to the fluorescence signal (Fig. 6A lower panels; see Discussion).

We quantified the AMPA-induced morphological changes of the spine cytoskeleton to determine the temporal profile and the magnitude of these events. When we measured the spine head area as a function of time, we observed a pronounced reduction of the spine size after AMPA treatment, with an average decay time of 67±12 s (N = 5 spines from 3 independent experiments) (Fig. 6B). Similarly, the bath application of AMPA caused a transfer of actin from the spine head into the dendritic shaft (Fig. 6C). Measuring the ratio of detected ABP-tdEosFP fluorophores in the spine head relative to those detected in the shaft, we saw a decay on a time scale of 120±70 s. These observations are in line with the depolymerization of F-actin during synaptic activity [31] and suggest that the reduction of the spine size precedes the loss of actin from the spine head into the dendritic shaft. Both effects were not observed in control neurons measured under baseline conditions.

We also conducted experiments using a lower AMPA concentration of 2 µM that had been reported to lead to rounding and the loss of motility of dendritic spines [31]. Whereas we observed the described rounding in some of the spines (Fig. 6D, arrow), the general trend was a reduction of the spine head size similar to that seen with 10 µM AMPA application (compare Fig. 6B and Fig. 6E). To probe whether the AMPA induced morphological changes affect the plasma membrane in the same way as the spine cytoskeleton, we performed dual PALM/QD imaging to simultaneously visualize the two structures at super-resolution (Fig. 6F). The quantification of these measurements shows that the membrane and the cytoskeleton after the 2 µM AMPA treatment remain closely related (mean distance 48±37 nm, N = 13) and not significantly different from the baseline measurements (P = 0.8, Mann-Whitney U test). This demonstrates that the spine membrane adapts dynamically to the changes of the spine morphology.

## Discussion

In this study, we have implemented PALM microscopy for long-term super-resolution dynamic imaging. The necessary tools and techniques are widely available, which makes this a relatively low-cost approach that may be easily set up in many laboratories using fluorescence microscopy.

We initially validated our experimental strategy by studying the organization of the actin cytoskeleton in dendritic spines in fixed neurons. From the high-resolution PALM images of mature dendrites it was possible to quantify morphological parameters of spines that are comparable with the results obtained by electron microscopy [23], [24]. We did not observe any correlations between the size of the spine head and the dimensions of the neck, in line with previous findings [23]. Upon closer inspection, the sub-spine distribution of actin is not homogeneous. The actin density within the spine head was generally higher in the center compared to the outer regions. These low-density regions appeared to consist of a filamentous mesh. The thickness of these structures is below the pointing accuracy of our detection and could thus not be determined accurately. However, it would be tempting to speculate that these represent actin filaments that have so far only been observed by electron microscopic analysis of the spine cytoskeleton [16], [17].

Subsequently, we performed live PALM experiments to observe the baseline dynamics of the spine morphology over tens of minutes. The quantification of the morphological spine parameters in living neurons is in very good agreement with the values obtained in fixed neurons, suggesting that the chemical fixation with paraformaldehyde did not affect the cytoskeletal structure noticeably. Furthermore, we observed that the temporal rearrangement of the spine cytoskeleton was more pronounced in the spine head and the length of the neck, while the width of the neck remained remarkably constant. This could reflect the fact that the actin filaments in the spine head are organized in a complex manner in contrast to the neck where the F-actin appears to have a linear organization [32]. Changes of the spine morphology may thus be expected to occur mainly in the head width and the length of the spine rather than the width of the neck structure. Since the neck represents a bottleneck to the diffusion of synaptic proteins and ions between the head and the shaft, the precise determination of its dimensions in live systems is important to understand its involvement in regulatory mechanisms [8]. We found that the neck opening (width of around 150 nm) occupies as little as 1% of the surface area of the spine head (mean diameter of 600 nm).

In this context it should be noted that our measurements refer to the dimension of the cytoskeleton as determined by the detection of the ABP-tdEosFP expression construct. In order to relate our findings to the localization of the plasma membrane, we combined PALM imaging with QD tracking of a diffusing membrane construct (GFP-GPI). This technique allowed us to visualize both, the actin cytoskeleton and the plasma membrane simultaneously at super-resolution. Our measurements showed a close correspondence between the cytoskeleton and the membrane, typically within a range of 50 nm. This distance includes the width of the plasma membrane (5–10 nm) in addition to the size of the antibodies (∼5 nm) and the streptavidin-coupled QDs (∼25 nm) used for labeling. The measured distances in the spine heads had a broader distribution compared to the neck. The likely cause of this difference is that F-actin filaments are distributed very unequally within the spine head and that consequently low density regions of the cytoskeleton at the periphery of the spine are sampled less efficiently than the core of the spine.

To provoke morphological changes of the spine cytoskeleton, we induced a sustained synaptic depolarization by bath application of AMPA and measured the concomitant alterations of the spine shape. We found that this treatment reproducibly led to the shrinkage of synaptic spines, followed by a reduction of the actin levels in spines. This is in agreement with the data by Halpain and colleagues, who found that glutamate receptor activation triggered a loss of F-actin and the collapse of the spines [33]. A different report has suggested that AMPA application causes a rounding of the spine heads and the loss of spine dynamics [31]. This difference may be explained by a concentration-dependence of the glutamatergic activation in the two studies (i.e. 10 µM versus 1–2 µM AMPA). We have therefore reassessed the effect of AMPA application at 2 µM and 10 µM concentration. While we occasionally observed the rounding of the spine head in response to glutamate receptor activation with 2 µM AMPA, the most consistent effect was the reduction of the size of the spines at either concentration, with or without the rounding effect. Our findings are thus suggestive of a depolymerization of the F-actin cytoskeleton during depolarization. In order to explore how the depolymerization of the actin cytoskeleton translates into changes of the position of the plasma membrane, we also performed dual-color PALM/QD tracking. We found that the correspondence between the two structures was equally close before and after the 2 µM application of AMPA. In other words, the position of the plasma membrane adapts dynamically to the rearrangement of the actin cytoskeleton.

In addition to the morphological changes, the levels of actin in the spine head decreased during AMPA application. This redistribution of actin could also be observed in the diffraction-limited images, where the fluorescence intensity of diffusing fluorophores contributes to the overall fluorescence. In contrast, in the PALM image reconstruction only fluorophores that are bound to F-actin are likely to be detected as well focused spots (with 2D-Gaussian shape) and thus represented in the image. Therefore, the decrease in the signal intensity of the spine head relative to the shaft is due not only to depolymerization of actin but also to an actual redistribution of actin through the spine neck. Since the observed redistribution of depolymerized actin from the spine head into the dendritic shaft occurred gradually and on a somewhat slower time scale, our findings also imply that the spine neck represents a barrier that limits the diffusion of actin monomers to the dendrite, and underlines the importance of the spine neck for the compartmentalization of the spine head as the site of synaptic neurotransmission.

The use of a low-affinity probe (ABP-tdEosFP) for live PALM imaging confers several beneficial features: 1) bleached fluorophores are replenished, in contrast to probes that are fused to the protein of interest, thereby enabling long-term recording of a given cellular structure; 2) the probe is not directly incorporated into the actin filaments, reducing the risk of altering the cytoskeletal organization; 3) the cell may tolerate higher levels of expression, meaning that there is a larger pool of photoactivatable probes. This also implies an effective increase of the sampling of the structure since a large number of binding sites are available for ABP-tdEosFP. Our experimental strategy also represents a step forward in comparison to the indirect measurements achieved by SPT techniques [14], [15], since we reconstruct the entire spine cytoskeleton itself at a relatively high temporal resolution. Our approach can therefore be used to study the long-term morphological rearrangement of actin during synaptic plasticity. Sub-diffraction dynamic imaging (20 s per frame) of dendritic spines in organotypic slices at less than 100 nm resolution was previously shown by Nägerl *et al.* using stimulated emission depletion microscopy (STED) [4]. However, in this study the authors made use of a diffusive cytosolic fluorophore (YFP) to visualize the shape of the spine, without resolving the internal organization of the cytoskeletal structure. Furthermore, in comparison to this approach we managed to reduce the high density of photons irradiating the sample by five orders of magnitude (from ∼400 MW/cm^2^ at 598 nm to ∼4 kW/cm^2^ at 561 nm) thus reducing the risk of phototoxicity. Indeed, we did not observe any consistent changes of any of the measured spine parameters or their dynamics over time (see Fig. 4C, movie S1).

In conclusion, our work brings together several innovative approaches to study the dynamics of cellular structures at super-resolution, namely long-term dynamic PALM imaging and the use of a low-affinity probe. Furthermore, we have combined live PALM imaging with QD-based single particle tracking in order to visualize two cellular structures (membrane and cytoskeleton) simultaneously at super-resolution. We believe that this novel approach is a powerful combination of techniques that can be applied to correlate single molecule dynamics with cellular structures at the nanometer scale, such as the movement of neurotransmitter receptors in relation to the postsynaptic density [34].

## Materials and Methods

### Cell culture and transfection

COS-7 cells (ATCC, cat. No. CRL-1651, [35]) were kept in DMEM medium (Invitrogen) supplemented with 10% fetal bovine serum (FBS), 2 mM glutamine and antibiotics (50 U/ml penicillin/50 µg/ml streptomycin) at 36°C and 5% CO_2_. For transfection, cells were grown on glass coverslips to a density of 2×10^4^/cm^2^ and transfected with FuGENE 6 (Roche) according to the manufacturer's protocol (2 µg DNA and 6 µg transfection reagent in 2 ml volume per 6 cm Petri dish). Cells were maintained for 24 h in culture medium containing 2% FBS and then fixed or used for live imaging (see FLAP experiments).

Rat primary hippocampal neurons were prepared as previously described [36], in accordance with the guidelines issued by the French Ministry of Agriculture and approved by the *Direction départamentale des services vétérinaires de Paris* (Ecole Normale Supérieure, Animalerie des Rongeurs, license B 75-05-20). All efforts were made to minimize animal suffering and to reduce the number of animals used. Briefly, hippocampi were dissected from Sprague Dawley rats at embryonic day 18, dissociated by trypsinization, plated at a density of 6×10^4^/cm^2^ on 18 mm glass coverslips that had been successively coated with 80 µg/ml poly-D,L-ornithine (Sigma) and 5% FBS (Invitrogen), and maintained in Neurobasal medium (Invitrogen) containing 2% B27 (Invitrogen) and 2 mM glutamine at 36°C/5% CO_2_. Neurons were generally transfected between day in vitro (DIV) 8 and 12 with Lipofectamine 2000 (Invitrogen) as suggested by the supplier (0.5 µg DNA and 2 µl transfection reagent in 600 µl volume per coverslip for 30 min). Mature hippocampal neurons were generally used for imaging experiments between DIV 20 and DIV 30.

### Expression constructs

The actin-binding sequence of ABP140 from *S. cerevisiae* (ABP, [20]; MGVADLIKKFESISKEE) was subcloned into the *Nhe* I/*Eco*R I restriction sites of the pDendra2-N vector (Clontech) using an overlapping primer pair (ctagccaccATGGGCGTGGCCGACCTGATCAAGAAGTTCGAGAGCATCAGCAAGGAGGAGactagtAgaatt). The coding sequence of tandem Eos fluorescent protein (tdEosFP, containing the T158R point mutation, [37]) was amplified by PCR (cDNA kindly provided by Mike Davidson) and shuttled into the ABP-containing construct with *Spe* I and *Not* I, yielding the pABP-tdEosFP expression construct. A photoactivatable mCherry-tagged β-actin construct was provided by Vladislav Verkhusha [29]. A GFP-tagged GPI expression construct was used to visualize the spine shape using QD-tracking [38].

### Fixation and immunocytochemistry

Cells were fixed in 0.1 M phosphate buffer (pH 7.4), containing 4% paraformaldehyde and 1% sucrose at 36°C for 10 min and rinsed in phosphate buffered saline (PBS, pH 7.4). The F-actin cytoskeleton in COS cells was labeled using 13 nM Alexa Fluor 647-phalloidin (Invitrogen, 1∶500 dilution) in PBS containing 1% bovine serum albumin (BSA, Sigma) for 1 h and washed in PBS. For Shank2 immunolabeling, neurons were post-fixed in methanol for 5 min at −20°C and blocked over night at 4°C in PBS containing 3% BSA. A polyclonal rabbit antibody directed against the PSD protein Shank2 (ProSAP1, [39]) was applied in blocking solution for 1 h at a dilution of 1∶500, followed by secondary Alexa Fluor 647-labeled donkey anti rabbit IgG (Invitrogen, 1∶500, 1 h). Fixed (labeled or unlabeled) COS-7 cells or neurons were imaged in PBS, after attaching multicolored 0.1 µm beads (TetraSpeck microspheres, Invitrogen) to the coverslips for drift correction (∼4×10^7^ beads in 20 µl PBS for 15 s, rinsed with PBS).

### Live imaging

Coverslips with rat hippocampal neurons were imaged in MEM medium without phenol red (Invitrogen) containing 2% B27, 2 mM glutamine, 1 mM pyruvate, 33 mM glucose, 20 mM HEPES, 1 µM tetrodotoxin (TTX) and 5 µM glycine ( = imaging buffer) at 35°C for durations of less than 1 h. AMPA receptors (AMPAR) were activated by bath application of 2 µM or 10 µM α-amino-3-hydroxy-5-methyl-4-isoxazolepropionic acid (AMPA). To correct for the x/y-drift of the microscope stage multicolored beads (TetraSpeck) were used as fiducial markers.

### Single molecule imaging

Super-resolution imaging was performed on an inverted Nikon Ti Eclipse microscope. Activation (405 nm/532 nm) and excitation (561 nm/641nm) lasers for PALM/dSTORM were combined in an external platform; the combined beam was expanded and re-collimated with a beam expander, directed through free air into the microscope and focused in the rear plane of a 100× objective (N.A. 1.49) using an achromatic converging doublet-lens with a focal length of 500 mm. Images were taken in wide-field configuration and, unless otherwise stated, the experiments were acquired under continuous illumination of both, activation and excitation laser light. Activation laser power was finely tuned during acquisition, in order to maintain the density of activated fluorophores constant; typical values of activation and imaging laser power densities on the sample were 7×10^−3^ to 3×10^−2^ kW/cm^2^ (405 nm), 4 kW/cm^2^ (561 nm), 1 kW/cm^2^ (532 nm), and 2.7 kW/cm^2^ (641 nm). Single-molecule tdEosFP signals were separated with a 561 nm dichroic (Di01-R561-25×36) and a single band 617 nm emission filter (FF01-617/73), expanded through a 1.5× lens in the tube-lens of the microscope and acquired with an Andor iXon EMCCD camera (512×512 pixels, pixel size 16 µm) at frame rates of 25 or 50 ms for up to 30 minutes. The low-resolution, conventional fluorescence images of the pre-converted form of tdEosFP were taken using a mercury lamp for illumination (excitation: Semrock FF01-485/20, emission: FF01-525/30).

To avoid a substantial degradation of the spatial resolution, sample drift needed to be corrected. The position along the optical axis (Z axis) was actively stabilized during the acquisition by the Nikon perfect focus system (PFS) integrated in the scope. Drift in the XY plane, which may be as high as 50–100 nm in 10 min, was corrected in the post-processing of the images using the multicolor beads described above as fiducial markers.

Dual-color dSTORM/PALM imaging was performed successively. First, we reversibly switched Alexa 647 fluorophores (labeled Shank2) between a dark state and a fluorescent state, under reducing buffer conditions (PBS, pH 7.4, containing 10% glucose, 50 mM β-mercaptoethylamine, 0.5 mg/ml glucose oxidase and 40 µg/ml catalase, degassed with N_2_; [26]) and continuous illumination of a 532 nm and a 641 nm lasers (dichroic: FF650-Di01, emission filter: FF01-684/24). Once a satisfactory density of single molecule detections had been achieved, we switched to PALM imaging of the tdEosFP fluorophores (tagged ABP).

Live dual-color measurements of the actin cytoskeleton and the spine membrane were done by combining PALM imaging of ABP-tdEosFP with QD-tracking of a GFP-GPI membrane expression construct with a Photometrics dual-view detection system, using the 561 nm laser as excitation for both, the converted form of tdEosFP and the QDs. Co-transfected neurons were labeled with a rabbit antibody directed against GFP (Synaptic Systems, 1∶10^4^ in imaging buffer, 4 min), followed by a secondary biotinylated anti-rabbit Fab fragment (Jackson ImmunoResearch, 1∶2000 in imaging buffer, 4 min) and streptavidin-coupled QDs emitting at 705 nm (Invitrogen Q10161MP, 1 nM in QD binding buffer, 1 min; see [40]). Note that these conjugated quantum dots are relatively large, with approximately 25 nm in diameter. The emitted light was then separated with a 633 nm dichroic and filtered (593/40 nm and 692/40 nm for tdEosFP and 705 nm QDs, respectively).

To quantify the fluorescence loss after photoactivation (FLAP), the 405 nm laser beam was focused in the center of the field of view, covering an area of ∼4 µm^2^. ABP-tdEosFP transfected COS-7 cells (in MEM imaging buffer at 35°C) were exposed to a short beam of the 405 nm laser, while images were taken every 30 s for 18 min using the mercury lamp (560/25 nm excitation and 614/70 nm emission filters).

### Image analysis

Raw images of single molecule signals were analyzed with an adapted version of the Multi-Trace Tracking MTT algorithm [41]. In practice, we run the algorithm developed by Serge *et al*. [41], toggling off its tracking features, and using the detection and fitting parts of the algorithm without further modification. For each individual frame, spatially separated point spread function (PSF) signals emitted by single fluorophores were detected and fitted with a 2D Gaussian distribution. Subsequently, super-resolution images were rendered by superimposing the position coordinates of the detected single molecules, represented with a 2D Gaussian curve with the same intensity value, using a standard deviation σ that had been previously determined by the localization accuracy of single fluorophores (typically 10 nm). In the case of live-cell PALM imaging, the number of frames used for reconstruction was chosen for the best compromise between temporal and spatial resolution, as described in the results section (data shown in Fig. 3C).

## Supporting Information

Figure S1 Sample images of dendritic protrusions, showing the morphological variety of spines described in [42], as seen by PALM imaging (A). In panel (B), neck length and width of dendritic spines show no correlation with the spine head diameter (for quantification see Fig. 2E).
(TIF)

Figure S2 Pointillist representation of live PALM imaging of a dendritic segment of an immature hippocampal neuron (DIV 9) at time 0 (black points), 12 min (blue) and 25 min (red) under continuous illumination and recording. The 405 nm laser power was continuously adjusted to yield a constant number of single molecule events. The chosen time-window for image reconstruction was 50 s (2000 frames). The data plotted in Fig. 3C were measured in the boxed region.
(TIF)

Movie S1 Live PALM imaging of a dendritic segment of a mature hippocampal neuron (DIV 27) under baseline conditions. The total length of the movie is 12.5 minutes. Each PALM frame was reconstructed from 2000 image frames of 25 ms, hence the temporal resolution is 50 s. The movie is rendered with a temporal sliding window of a step of 2.5 s. The width of the field of view is 12 µm. The drift of the movie was corrected with fiducial markers, therefore the actual movement of the dendrite is visualized.
(AVI)

Movie S2 Simultaneous PALM imaging and QD tracking. ABP-tdEosFP (top) and QD signals (bottom) were simultaneously imaged in a hippocampal neuron at DIV 27. The detected ABP-tdEosFP fluorophores were used to reconstruct the actin cytoskeleton of the dendritic spine (cumulative movie, middle panel); the QD trajectory (bottom) served to outline the shape of the spine membrane. Field of view is 5 µm×3 µm.
(AVI)

Movie S3 Live PALM imaging of a large dendritic segment of a mature hippocampal neuron (DIV 28), treated with 10 µM AMPA, as indicated in the movie. Movie rendered with a sliding window of 2000 frames of 25 ms ( = 50 s) and a step of 2.5 s. Field of view, 35 µm×16 µm.
(AVI)

Movie S4 Movie of an individual spine during 10 µM AMPA treatment (detail from Movie S3, see also Fig. 6). Field of view, 8 µm×5 µm.
(AVI)
