# Supplementary figures and images for: Super-Resolution Dynamic Imaging of Dendritic Spines Using a Low-Affinity Photoconvertible Actin Probe
Source: PLoS One. 2011 Jan 17;6(1):e15611. doi: 10.1371/journal.pone.0015611 (PMC3022016; doi:10.1371/journal.pone.0015611)

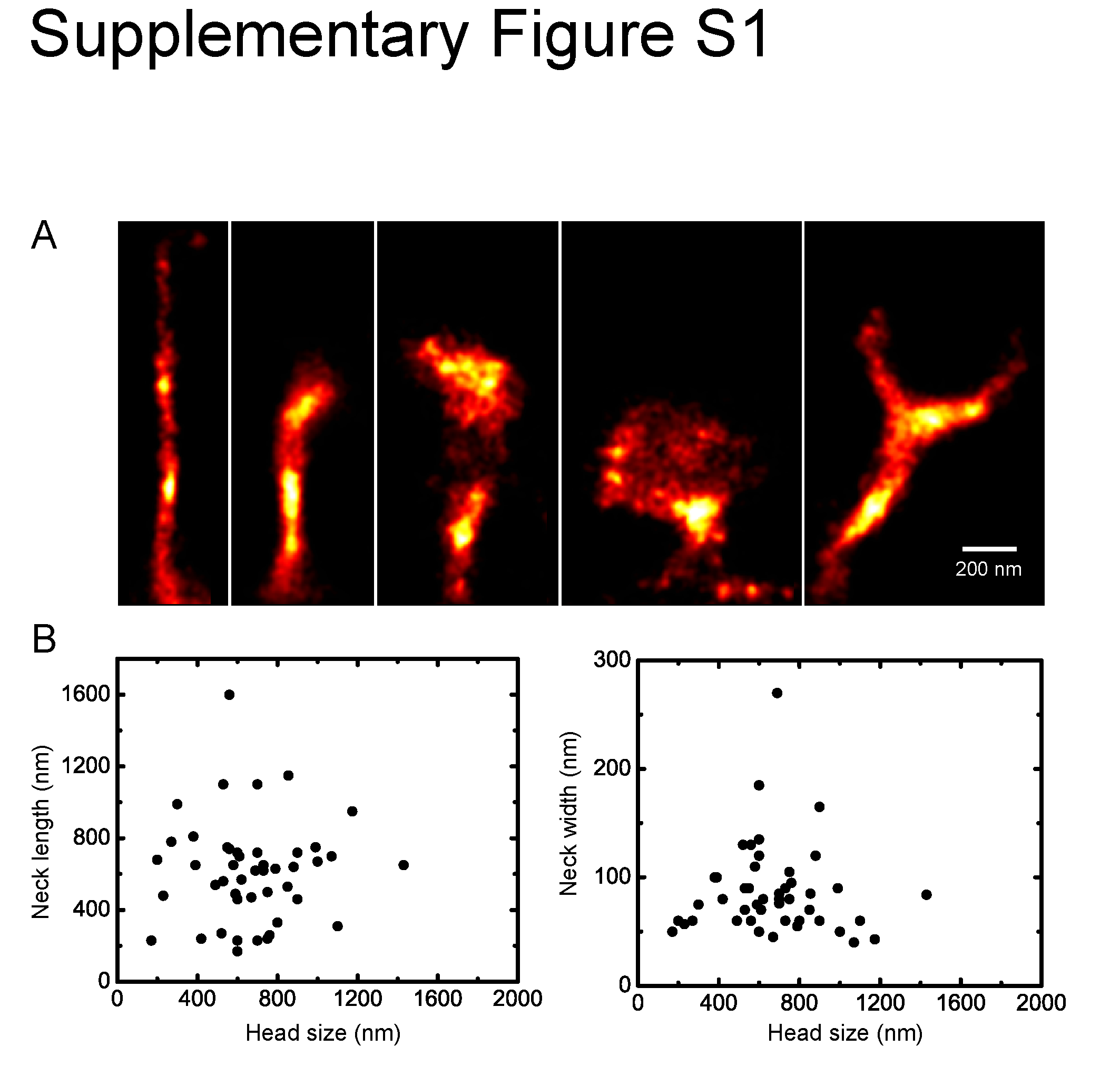

Supplement: Figure S1 — Sample images of dendritic protrusions, showing the morphological variety of spines described in [42], as seen by PALM imaging (A). In panel (B), neck length and width of dendritic spines show no correlation with the spine head diameter (for quantification see Fig. 2E). (TIF) [file pone.0015611.s001.tif]

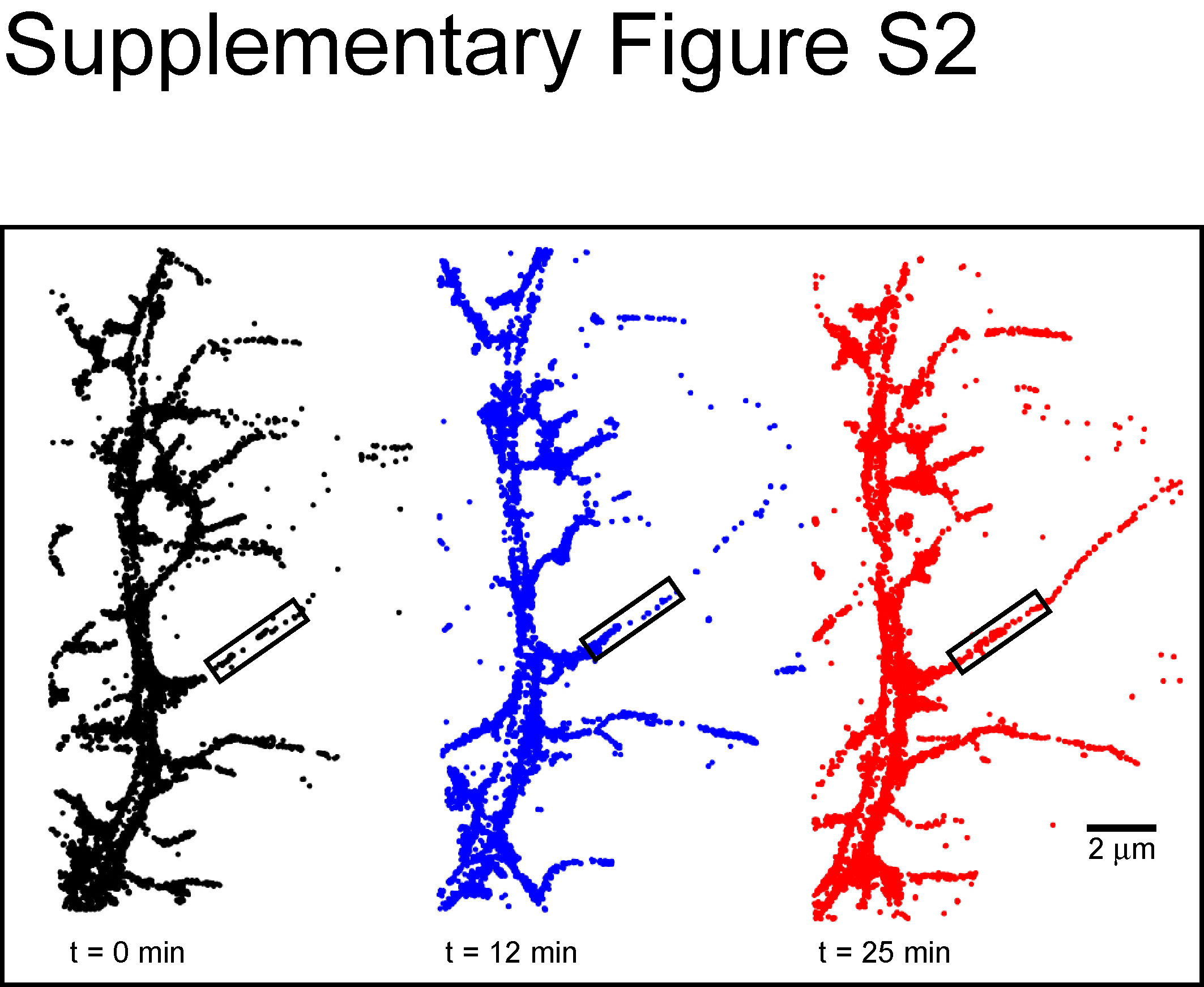

Supplement: Figure S2 — Pointillist representation of live PALM imaging of a dendritic segment of an immature hippocampal neuron (DIV 9) at time 0 (black points), 12 min (blue) and 25 min (red) under continuous illumination and recording. The 405 nm laser power was continuously adjusted to yield a constant number of single molecule events. The chosen time-window for image reconstruction was 50 s (2000 frames). The data plotted in Fig. 3C were measured in the boxed region. (TIF) [file pone.0015611.s002.tif]
